# Pre-pubertal exposure with phthalates and bisphenol A and pubertal development

**DOI:** 10.1371/journal.pone.0187922

**Published:** 2017-11-20

**Authors:** Monika Kasper-Sonnenberg, Jürgen Wittsiepe, Katharina Wald, Holger M. Koch, Michael Wilhelm

**Affiliations:** 1 Department of Hygiene, Social and Environmental Medicine, Ruhr-University Bochum, Bochum, Germany; 2 Institute for Prevention and Occupational Medicine of the German Social Accident Insurance, Institute of the Ruhr-University Bochum, Bochum, Germany; Universidad Miguel Hernandez de Elche, SPAIN

## Abstract

**Objective:**

Epidemiological studies indicate associations between childhood exposure with phthalates and bisphenol A (BPA) and the pubertal development. We examined associations between the pre-pubertal phthalate and BPA body burden and the longitudinally assessed sexual maturation of eight- to thirteen-year-old children.

**Methods:**

We started with eight- to ten-year-old children in the baseline study and quantified phthalate metabolites and BPA in 472 urine samples (250 boys; 222 girls; mean age: 8.8 years). Associations between the pubertal development, assessed in three annual follow-up studies by Puberty Development scale questionnaires (PD scales), and the chemical exposure from the baseline visit were longitudinally analyzed with generalized estimation equations.

**Results:**

The number of children with both chemical measures and PD scores (calculated from the PD scales) was 408. In the third follow-up, 49% of the girls and 18% of the boys had reached mid-puberty. For girls, we observed a delayed pubertal development with the di-hexyl-ethyl phthalate (DEHP) metabolites (β: -0.16 to -0.23; p ≤ 0.05 or p ≤ 0.1), mono-n-butyl phthalate (β: -0.15; 95% CI: -0.31; 0.01), mono-benzyl phthalate (β: -0.11; 95% CI: -0,24; -0,01), and mono-ethyl phthalate (MEP) (β: -0.15; 95% CI: -0.28; -0.01). In addition, significant non-linear associations of the DEHP metabolites and BPA with the PD scores were found, when their quadratic effects were included in the GEE models. In boys, no consistent relationships between the PD scores and the chemicals were detected except of an accelerated development with the ∑DEHP metabolites (β: 0.16; 95% CI: -0.02; -0.34).

**Conclusion:**

We found indications that pre-pubertal exposures with phthalates and BPA were associated with pubertal timing in children, particularly in girls. For boys, associations were inconsistent, and not necessarily in line with the known anti-androgenicity of some phthalates during prenatal exposure.

## Introduction

In puberty, hormones regulate the final maturation of the body systems. Pre-puberty is a key period in the path of the body’s maturation as well as a potentially susceptible window to chemical exposures. Puberty dysregulation may result in increased risk of disease in adult life (reviewed in [1]). Estrogen/androgen balance is a key component in sex-specific tuning of the whole puberty process, interplaying with growth factors in order to regulate growth and maturation of all organs and systems during puberty. Accordingly, exposure to endocrine disruptors may substantially alter the puberty process [1].

Phthalates and bisphenol A (BPA) are used in a variety of plastic materials, such as polyvinyl chloride (e.g. floor carpeting, cables) but also in personal care products, medical devices, and epoxy resins. Thus, they are widely distributed in the environment, our daily surroundings and contaminated foodstuff. The chemicals can enter the body by ingestion or skin contact, but also by inhalation, e.g. of contaminated house dust [2]. Several epidemiological studies and animal experiments identified phthalates and BPA as endocrine disrupting chemicals (EDC’s) [3–6]. These are a heterogeneous ensemble of substances that can interfere with the function of the endocrine systems through diverse mechanisms, such as agonism/antagonism with nuclear receptors (NRs), inhibition of the hormone biosynthesis and interference with the hypothalamic-pituitary-gonadal/thyroid/adrenal (HPG/T/A) axis [1, 7]. Exposures with EDC’s are highly plausible risk factors for altered puberty onset [8]. More recent findings reveal a link of phthalate and BPA exposure to the development of children, e.g. pubertal development, changes of the immune response, or the development of asthma [4, 9, 10, 11]. Epidemiological data indicate associations between phthalate and BPA exposure and pubertal timing but results are inconsistent. Some studies observed a delayed and others an accelerated development [9, 11, 12, 13, 14].

Therefore, we measured phthalate metabolites and BPA in eight- to ten-year-old children from the German Duisburg Birth and Bochum Cohort studies and analyzed relationships with the timing of puberty in three annual follow-up studies.

## Material and methods

### Study design and participants

**Fig 1:**
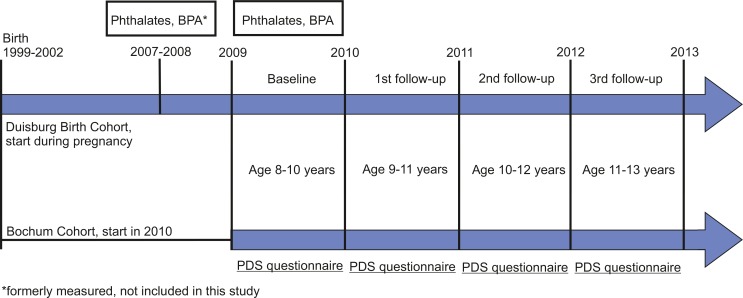
Timeline of the cohort studies.

The study was carried out in Duisburg and Bochum, Germany. Both cities are located in the Ruhr District of North Rhine-Westphalia. The first enrollment of participants was completed in 2002 for Duisburg (birth cohort study). Initially, 232 pregnant mothers from Duisburg responded to the study. During this time, the study focused on the human biomonitoring of polychlorinated dibenzo-p-dioxins and dibenzofurans (PCDD/Fs), polychlorinated biphenyls (PCBs) and organochlorine pesticides [15]. Since 2006, we started new follow-up studies focusing on the human biomonitoring of further chemicals with endocrine disrupting properties. In 2010, we recruited 359 additional mother-child pairs from Bochum (cohort study). The children from both cohorts were born between 1999 and 2002. Details of the study design were published previously [16]. In the new baseline study (2009–2010) we collected urine samples and conducted three annual follow-up studies (years 2010–2011; 2011–2012; 2012–2013) to monitor the pubertal development. The children’s age was between eight and thirteen years over the four included study periods. The distance between each follow-up was approximately one year for each participant (Fig 1).

The study was reviewed and approved by the Ethical Commission of the Medical Facilities at Ruhr-University Bochum, Germany (Registry no. 1478; 3220–08; 3486–09). We collected written informed consent from each participating child as well as their parents.

### Samplings

First morning urine samples were collected in the baseline study (year of enrollment: 2009–2010) from 472 children (Duisburg: 62 boys; 52 girls; mean age: 8.5; range 8–9 years; Bochum: 186 boys; 172 girls; mean age: 8.9; range 8–10 years).

### Chemical analyses

BPA and 21 phthalate metabolites (MEHP, OH-MEHP, oxo-MEHP, cx-MEHP, OH-MiNP, oxo-MiNP, cx-MiNP, OH-MiDP, oxo-MiDP, cx-MiDP, MEP, MMP, MBzP, MCHP, MnPeP, MnOP–the full names are provided in S1 Table) representing eleven parent phthalates in 472 urine samples were determined by multidimensional liquid chromatography coupled to tandem mass spectrometry (LC/LC–MS/MS) using isotope-labeled standards. After collection, all samples were stored at -20°C until the fieldwork was completed and were then simultaneously measured. Concentrations below the limits of quantification (LOQ) were set to one-half of the LOQ. Details of the analytical methods were published elsewhere [17–19]. The detection rates of 18 phthalate metabolites and BPA ranged between 81 to 100%. For the three metabolites MCHP, MnPeP and MnOP the detection rates were only 16.7, 7.4 and 1.5%. Therefore, we disregarded these metabolites in the further analyses. We calculated summary measures of the phthalates with two or more measured metabolites (∑DEHP, ∑DiNP, ∑DiDP, ΣDnBP, ΣDiBP) by summation of the volume-related concentrations (μg/l) of their respective metabolites. Five urines with creatinine concentrations below 30 mg/dl and two urines above 300 mg/dl were excluded from the further analysis (using these concentrations as indicators for too diluted or too concentrated urines). Therefore, the resulting samples size was N = 465.

### Collection of anthropometric measures and further information

In each of the four visits, trained staff measured body height and weight of the participating children and collected further information (e.g. age, socio-demographic and life style information) with standardized questionnaires.

### Pubertal development scales (PD scales)

We recorded the pubertal development of the children by self-reporting using the German version of the “Pubertal Development Scales–PD scales” questionnaire (children’s version) [20], developed by Petersen et al. [21]. The questionnaire data were collected in four examination periods: baseline study (age range: 8–10 years); first follow-up (age range: 9–11 years); second follow-up (age range: 10–12 years); third follow-up (age range: 11–13 years) with a distance of approximately one year between the follow-up examinations for each child. The questionnaire contained five items (PD scales), coded on a four-level ordinal response scale, regarding the following physical puberty markers: growth spurt, skin changes, pubic hair growth for both sexes; breast development and menarche onset for the girls; facial hair growth and voice change for the boys (S2 Table). In awareness of embarrassment of the children, we provided them a separate room during the field studies. There, they completed the questionnaire without the risk of being watched by a field worker or their chaperon(s). Afterwards, the children placed it in an envelope and handed it out to the field workers.

**PD scores** were calculated from two or three items, respectively (for girls: pubic hair growth and breast development; for boys: pubic hair growth, facial hair growth, and voice change) by summarizing the values from the given answers according to Carskadon and Acebo [22], resulting in ordinal PD scores. Pubertal stages (pre-pubertal, early puberty, mid-puberty, late puberty, post-pubertal) were also calculated from these items and, for girls, by additionally using the information of having onset of menarche [22].

### Statistical analyses

Out of 478 PD scales questionnaires collected in the baseline study, 68 questionnaires dropped out because the children did not participate in the follow-up studies. Most missing data of the PD scales throughout the four examinations occurred when the child either did not complete the questionnaire or was unable to answer the question (item: I do not know). Because we detected some overestimations of the developmental process (e.g. a child answered on a higher scale one or two years earlier, especially in the baseline study) we also set these values to missing value. These missing data were imputed by multiple ordinal regression analyses adjusted for sex, age and body mass index (BMI) due to the following criteria: a minimum of two complete data sets of the PD scale questionnaire was available, and there was no missing information of the age and body measures (height, weight). First *a posteriori* likelihoods of the PD scales were predicted. Secondly, missing values were randomly drawn according to the *a posteriori* likelihoods. In this manner, we received 410 completed questionnaires from each of the four visits compared to 478, 394, 362 and 300 questionnaires prior to the imputation procedure. Internal validity and reliability of the PD scales before and after imputation was tested by the Cronbach’s alpha test (S3 Table). The test measures the internal consistency of an empirical investigation and provides information on how exact the construct (e.g. pubertal development) can be measured. It provides dependent from the number of items used the average inter-item-association. This means, the higher the correlation coefficient is, the higher are the inter-item-association and the reliability of the construct [23].

Imputations and Cronbach’s alpha were calculated with the open source software R, version 3.1.2 (R Foundation for Statistical Computing).

The associations between the PD scores from the three follow-up studies and the ln-transformed chemical concentrations from the baseline study were longitudinally analyzed by multinomial and binomial generalized estimating equations (GEE). This procedure was suitable for analyzing longitudinal data in order to assess an average population-based effect of an influencing factor on a dependent variable (here: PD scores and single PD scales). A repeated measure within one subject will not be independent from each other and therefore, we allowed for considering autocorrelation by introducing a correlation structure with a first-order autocorrelation as a basis. A linear development of puberty over time was also taken as given. This means that the outcome variable at a time t was linearly dependent on the outcome variable at the time t-1.

The GEE models were also performed using the non-imputed original PD scores and additionally the single PD scales (definitely started and higher: ≥ 3) (S4 Table). For voice change and facial hair growth in boys, we had to perform a binary logistic regression analysis from the data of the third follow-up study because these events were only positive in the third follow-up and therefore, could not be longitudinally analyzed. We further tested non-linear influences of the exposure data on the PD scores by generalized additive models (GAM) and subsequently accounted for them, if present, in the GEE regression models by including the quadratic term of the ln-transformed exposure variables (MEHP*MEHP; cx-MEHP*cx-MEHP; BPA*BPA) (according the SAS Global Forum publication no. 378 [24]). We also conducted regression models, controlling for co-exposure with the sum of the metabolites of the high molecular weight (HMW) phthalates ∑DEHP, ∑DiNP, ∑DiDP, and BPA for the analyses of the low molecular weight (LMW) phthalate metabolites; and co-exposure with the metabolites MEP, MMP and BPA for the analyses of HMW phthalate metabolites. For this purpose, we included these variables in the multivariate analyses as covariates. Because most phthalate metabolite concentrations were highly correlated, the selection of the metabolites for these models followed lower correlation coefficients between the metabolites with coefficients of around 0.4 or smaller (S5 Table).

Each statistical analysis was stratified by sex. The covariates age, BMI and the urinary creatinine concentrations (as recommended in [25]) were included in the multiple regression models.

The regression coefficients (β) were presented together with their corresponding 95% confidence intervals (95% CI) and, in case of the logistic regression and binomial GEE analyses, with their Odds Ratios (OR’s) and 95% CI’s. A p-value ≤ 0.05 was considered as significant, if not otherwise stated. This part of the statistical analyses was performed with the statistical software SAS, version 9.4 SAS Institute, Cary, NC, USA.

## Results

After excluding too diluted or too concentrated urine samples (creatinine should be between 0.3 and 3.0 g/L), 465 urine samples remained. Among the 410 children with questionnaires throughout the four study periods, 408 children provided both questionnaire data and applicable urine samples (210 boys; 198 girls). The BPA and phthalate metabolite concentrations in urine samples of the eight- to ten–year-old children were similar as for the general population in Germany and Europe (background levels), and were not significantly different between boys and girls as previously published [16] (Table 1).

**Table 1:** Characteristics of the study population and the phthalate metabolite and BPA concentrations in urine.

	Boys (N = 210)	Girls (N = 198)
**Age (years)**	**AM (SD)**	
Baseline study	8.8 (0.5)	8.7 (0.5)
First follow-up	9.7 (0.5)	9.6 (0.5)
Second follow-up	10.7 (0.5)	10.7 (0.5)
Third follow-up	11.9 (0.5)	11.8 (0.5)
**PD scores**	**Median (range)**	
Baseline study	3 (3–6)	2 (2–5)
First follow-up	3 (3–6)	2 (2–6)
Second follow-up	3 (3–6)	3 (2–6)
Third follow-up	4 (3–8)	4 (2–7)
**BMI (kg/m**^**2**^**)**	**AM (SD)**	
Baseline study	17.2 (2.1)	17.0 (2.3)
First follow-up	17.8 (2.4)	17.5 (2.5)
Second follow-up	17.7 (2.5)	17.5 (2.8)
Third follow-up	18.9 (2.8)	18.6 (2.9)
**Phthalate metabolites/BPA (μg/l) (baseline study)**	**GM (95% CI)**	
∑4DEHP^a^	74 (68–82)	74 (66–82)
∑3DiNP^b^	31 (28–35)	32 (28–36)
∑3DiDP^c^	4.0 (3.6–4.4)	3.9 (3.5–4.3)
∑2DiBP^d^	63 (57–70)	69 (62–78)
∑2DnBP^e^	48 (44–54)	50 (45–55)
MEP	25 (22–28)	25 (22–29)
MBzP	7.3 (6.4–8.4)	6.2 (5.4–7.2)
MMP	3.5 (2.9–4.2)	2.7 (2.3–3.3)
BPA	2.1 (1.9–2.3)	2.1 (1.9–2.5)

AM: arithmetic mean; SD: standard deviation; GM: geometric mean; 95% CI: 95% confidence interval of GM.

a: ∑MEHP+ OH-MEHP+ oxo-MEHP+cx-MEHP

b: ∑OH-MiNP+oxo-MiNP+cx-MiNP

c: ∑OH-MiDP+oxo-MiDP+cx-MiDP

d: ∑MiBP+OH-MiBP

e: ∑MnBP+OH-MnBP—all in μg/l

Calculating the pubertal stages according to Carskadon et Acebo [22], we detected that girls developed puberty earlier than boys did. For instance, 7.6% of the girls and 1.0% of the boys estimated themselves to mid-puberty (stage 3) in the baseline study. In the third follow-up 49% of the girls but only 18% of the boys were in the mid-pubertal stage 3 (Table 2). In the third follow-up 18% girls (N = 35) reported menarche onset with a mean age of 11.3 ± 0.9 years. Post puberty (stage 5) was not reported during our study periods.

**Table 2:** Pubertal development in boys and girls.

		Pubertal stage (%)^a^^,^^b^				PD scales (N)				
Age (years)		Stage 1	Stage 2	Stage 3	Stage 4	Me-narche onset (yes)	Breast deve-lop-ment^c^	Pubic hair ^c^	Facial hair ^c^	Voice change^c^
**8–10** **(baseline)**	**Boys**	88	10	1	0	—	—	0	0	1
	**Girls**	78	13	7.6	1	2	3	3	—	—
**9–11** **(first follow-up)**	**Boys**	81	18	0.5	0	—	—	1	0	0
	**Girls**	58	23	18	1	2	7	9	—	—
**10–12** **(second follow-up)**	**Boys**	61	34	4.7	0	—	—	11	0	0
	**Girls**	30	25	39	5.6	11	28	26	—	—
**11–13** **(third follow-up)**	**Boys**	38	45	18	0	—	—	34	18	5
	**Girls**	15	19	49	18	35	56	59	—	—

Stage 1: pre-pubertal; stage 2: early puberty; stage 3: mid-puberty; stage 4: late puberty

a: frequencies of pubertal stages (%), calculated from the partly imputed PD scales (N = 408)

b: staging according to [22]

c: frequencies of the PD scales when the answer was “definitely started = 3” or higher (N), calculated from the partly imputed PD scales (N = 408)

**Fig 2:**
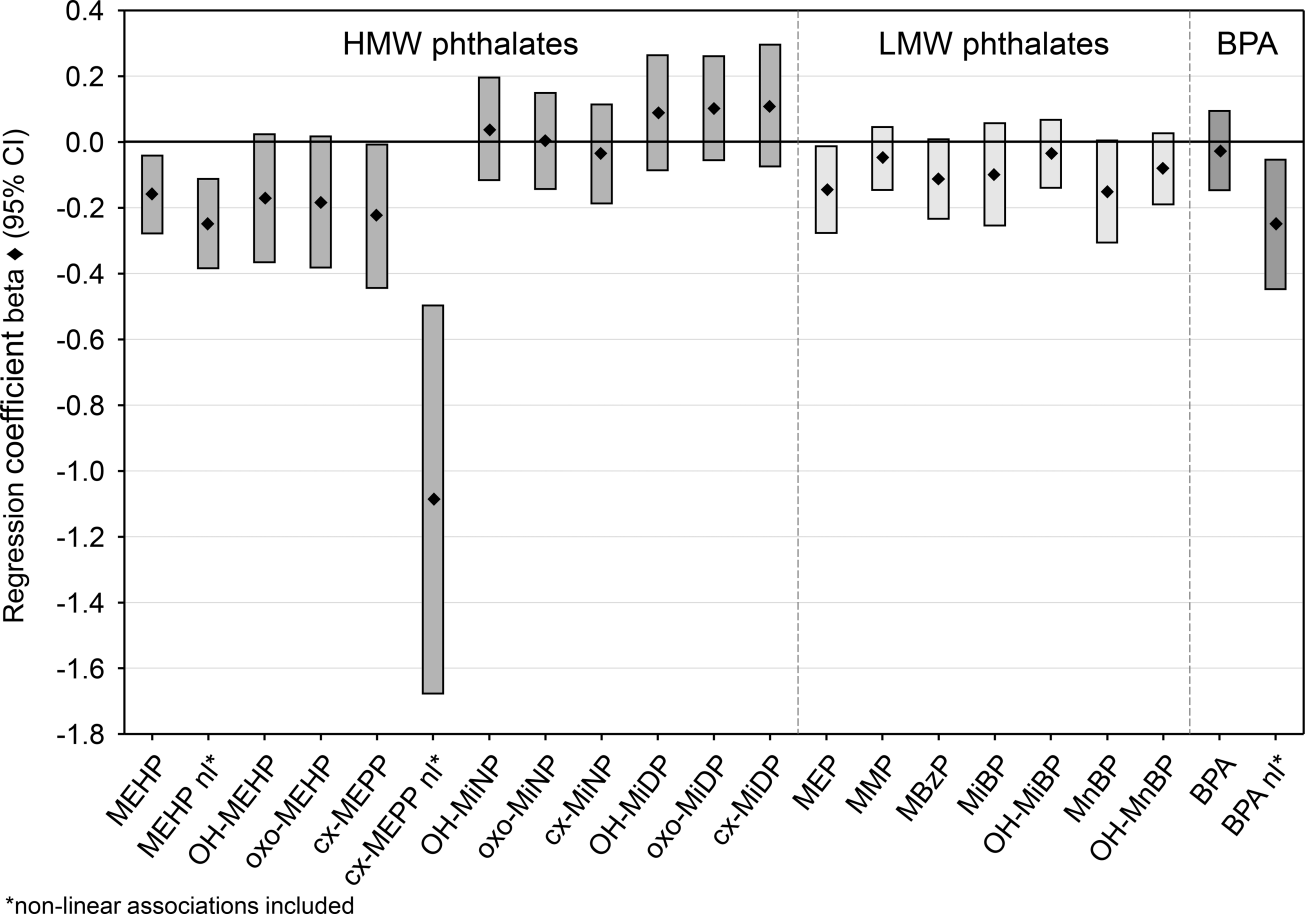
Regression coefficients and 95% confidence intervals of associations between the PD scores and the exposures with phthalate metabolites and BPA in girls. (HMW = high molecular phthalates; LMW = low molecular weight phthalates); adjusted for age (years), BMI (kg/m2), creatinine (mg/dl).

In most cases, we detected negative associations of the phthalate metabolite and BPA levels with the PD scores in girls, pointing to a delayed pubertal development, except for the DiDP and DiNP metabolites (Fig 2; Table 3). The strongest effects were observed with the four DEHP metabolites, and MEP, MnBP and MBzP. The associations were also seen in the models when additionally adjusting for HMW phthalates and BPA (association with LMW phthalates) and for MEP, MMP and BPA (association with HMW phthalates). In general, the percent changes on the average PD scores from the three follow-up studies (arithm. mean: 3.35 ± 1.15) were below ± 10%. For instance, the single phthalate metabolite model predicted 4.8% to 6.8% lower PD scores with the DEHP metabolites and 4.3% lower PD scores with MEP, whereas 2.8% to 3.0% higher PD scores were detected for the DiDP metabolites (Table 3). Furthermore, we found significant non-linear relationships between the MEHP, cx-MEPP and BPA concentrations with the PD scores. When accounting for these effects in the GEE models, the negative associations became stronger; for MEHP by a factor of approximately 1.6, for cx-MEPP 4.7, and eight for BPA with 7.4%, 32.5% and 7.5% lower PD scores for MEHP, cx-MEPP and BPA, respectively (Table 3). The BMI was not associated with higher metabolite concentrations as already published by Kasper-Sonnenberg et al. [16], but was positively associated with the PD scores specifically in girls, reflecting the important role of the body weight in the pubertal development.

**Table 3:** Multinomial GEE analyses of the PD scores with phthalate metabolites and BPA concentrations (ln-μg/l) in girls (N = 198).

Single phthalate metabolite models					Controlled for multiple phthalate metabolites				
	β	95% CI		% Change^c^		β	95% CI		% Change^c^
**MEP**	**-0.145**^**b**^	**-0.280**	**-0.010**	**-4.33**	**MEP**^**d**^	**-0.118**^**a**^	**-0.257**	**0.020**	**-3.52**
**MMP**	-0.050	-0.149	0.048	-1.49	**MMP**^**d**^	-0.064	-0.165	0.036	-1.91
**MBzP**	**-0.113**^**a**^	**-0.236**	**0.011**	**-3.37**	**MBzP**^**d**^	-0.096	-0.238	0.047	-2.87
**MiBP**	-0.098	-0.257	0.060	-2.93	**∑DiBP**^**d**^	-0.022	-0.197	0.153	-0.66
**OH-MiBP**	-0.036	-0.143	0.070	-1.07					
**MnBP**	**-0.151**^**a**^	**-0.309**	**0.008**	**-4.51**	**∑DnBP**^**d**^	-0.106	-0.292	0.079	-3.16
**OH-MnBP**	-0.082	-0.193	0.029	-2.45					
**MEHP**	**-0.160**^**b**^	**-0.281**	**-0.039**	**-4.78**	**∑DEHP**^**e**^	**-0.195**^**a**^	**-0.409**	**0.017**	**-5.82**
**OH-MEHP**	**-0.171**^**a**^	**-0.369**	**0.027**	**-5.10**					
**oxo-MEHP**	**-0.183**^**a**^	**-0.385**	**0.020**	**-5.46**					
**cx-MEPP**	**-0.226**^**b**^	**-0.447**	**-0.005**	**-6.75**					
**OH-MiDP**	0.089	-0.089	0.266	2.66	**∑DiDP**^**e**^	0.142	-0.060	0.344	4.24
**oxo-MiDP**	0.102	-0.059	0.264	3.04					
**cx-MiDP**	0.111	-0.078	0.299	3.31					
**OH-MiNP**	0.039	-0.120	0.198	1.16	**∑DiNP**^**e**^	0.021	-0.156	0.199	0.63
**oxo-MiNP**	0.003	-0.146	0.152	0.09					
**cx-MiNP**	-0.037	-0.190	0.117	-1.10					
**BPA**	-0.027	-0.150	0.097	-0.81					
**Non-linear associations included**									
**MEHP**	**-0.249**^**b**^	**-0.387**	**-0.110**	**-7.43**					
**cx-MEPP**	**-1.087**^**b**^	**-1.680**	**-0.494**	**-32.45**					
**BPA**	**-0.251**^**b**^	**-0.451**	**-0.051**	**-7.49**					

95% CI: 95% confidence interval; β: regression coefficient

a: p ≤ 0.1

b: p ≤ 0.05

c: calculated from the average PD score (mean) from 3 follow-up studies = (beta/mean)*100

Single pollutant models adjusted for

BMI (kg/m^2^), age (years), creatinine (mg/dl)); a: p ≤ 0.1; b: p ≤ 0.05.

Controlled for multiple phthalate metabolites

d: LMW phthalates: adjusted for HMW phthalates (∑DEHP, ∑DiDP, ∑DiNP, BPA (all: ln-μg/l)), BMI (kg/m^2^), age (years), creatinine (mg/dl)

e: HMW phthalates: adjusted for LMW phthalates (MEP, MMP, BPA (all: ln-μg/l), BMI (kg/m^2^), age (years), creatinine (mg/dl).

In boys, we did not observe consistent associations between the PD scores and the phthalate metabolites or BPA except for an accelerated development with MEHP (β: 0.13; 95% CI: -0.03; -0.24) and cx-MEPP (β: 0.16; 95% CI: -0.03; -0.35) (Table 4). For the LMW phthalates, almost only negative associations between the PD scores and the metabolites were seen, whereas for the HMW phthalates there were only positive associations. Generally, the percent changes on the average PD scores from the three follow-up studies (arithm. mean; 3.7 ± 0.9) were below 5%. For instance, the model predicted 4.3% higher PD scores with ∑DEHP and 0.4% to 0.8% lower PD scores with MMP, MBzP and the DiBP metabolites MiBP and OH-MiBP (Table 4). We also tested for non-linearity of the metabolites on the PD scores, but parameter estimates did not considerably change. Therefore, we did not perform an enhanced analysis with possible quadratic effects of the metabolites.

**Table 4:** Multinomial GEE analyses of the PD scores with phthalate metabolites and BPA concentrations (ln- μg/l) in boys (N = 210).

Single phthalate metabolite models					Controlled for multiple phthalate metabolites				
	β	95% CI		% Change^c^		β	95% CI		% Change^c^
**MEP**	0.032	-0.111	0.174	0.87	**MEP**^**d**^	-0.005	-0.150	0.139	-0.14
**MMP**	-0.016	-0.108	0.077	-0.43	**MMP**^**d**^	-0.037	-0.131	0.056	-1.00
**MBzP**	-0.023	-0.163	0.117	-0.62	**MBzP**^**d**^	-0.140	-0.311	0.031	-3.79
**MiBP**	-0.030	-0.191	0.132	-0.81	**∑DiBP**^**d**^	-0.147	-0.364	0.07	-3.98
**OH-MiBP**	-0.024	-0.131	0.083	-0.65					
**MnBP**	0.040	-0.145	0.224	1.08	**∑DnBP**^**d**^	-0.083	-0.313	0.147	-2.25
**OH-MnBP**	0.005	-0.125	0.134	0.14					
**MEHP**	**0.130**^**b**^	**0.025**	**0.236**	**3.52**	**∑DEHP**^**e**^	**0.159**^**a**^	**-0.018**	**0.335**	**4.30**
**OH-MEHP**	0.122	-0.047	0.291	3.30					
**oxo-MEHP**	0.137	-0.038	0.313	3.71					
**cx-MEPP**	**0.162**^**a**^	**-0.026**	**0.35**	**4.39**					
**OH-MiDP**	0.118	-0.037	0.273	3.19	**∑DiDP**^**e**^	0.144	-0.034	0.322	3.90
**oxo-MiDP**	0.096	-0.071	0.264	2.60					
**cx-MiDP**	0.128	-0.051	0.307	3.47					
**OH-MiNP**	0.050	-0.055	0.156	1.35	**∑DiNP**^**e**^	0.061	-0.093	0.214	1.65
**oxo-MiNP**	0.045	-0.093	0.183	1.22					
**cx-MiNP**	0.029	-0.133	0.192	0.79					
**BPA**	0.092	-0.045	0.229	2.49					

95% CI: 95% confidence interval; β: regression coefficient

a: p ≤ 0.1

b: p ≤ 0.05

c: calculated from the average PD score (mean) from 3 follow-up studies = (beta/mean)*100; Single pollutant models adjusted for: BMI (kg/m^2^), age (years), creatinine (mg/dl)) Controlled for multiple phthalate metabolites:

d: LMW phthalates: adjusted for HMW phthalates (∑DEHP, ∑DiDP, ∑DiNP, BPA (all: ln-μg/l)), BMI (kg/m^2^), age (years), creatinine (mg/dl)

e: HMW phthalates: adjusted for LMW phthalates (MEP, MMP, BPA (all: ln-μg/l), BMI (kg/m^2^), age (years), creatinine (mg/dl)

When calculating the regression models with the original non-imputed PD scores we detected similar results for boys and girls (S4 Table) compared with the partly imputed data sets, but statistical power was reduced due to the smaller sample size.

We conducted additional longitudinal binomial analysis using the single PD scales (event has definitely started or higher) that are included in the calculation of the PD scores.

For girls: Menarche onset (yes vs. no) was not integrated in the calculation of the PD scores but we decided to use this item also for the longitudinal analyses of the single PD scales. Almost all LMW metabolites were negatively associated with the single PD scales for breast development, pubic hair growth and menarche onset. The DEHP metabolites were negatively associated with breast development (OR’s 0.69–0.81), pubic hair growth (OR’s 0.72–0.86) and menarche onset (OR’s 0.70–0.82). Exposure with the DiDP metabolites was positively associated with breast development (OR’s 1.13–1.81) and pubic hair growth (OR’s 1.40–1.65) but negatively with menarche onset (OR’s 0.74–0.82). The DiNP metabolite levels were almost all positively associated with breast development (OR’s 1.15–1.31) and menarche onset (OR’s 1.08–1.14) and more or less unrelated with pubic hair growth (OR’s 0.98–1.07). Taken together, we found similar associations when using the single PD scales compared with the overall PD scores. Breast development and menarche onset appeared to be more affected by the metabolite concentrations than pubic hair growth. BPA showed no consistent relationship with the single PD scales (Table 5).

**Table 5:** Binomial GEE analyses of single PD scales with phthalate metabolites and BPA concentrations (ln- μg/l) in girls (N = 198).

	Breast development (yes vs. no)^c^			Pubic hair growth (yes vs. no)^c^			Menarche onset (yes vs. no)^d^		
	OR	95% CI		OR	95% CI		OR	95% CI	
**MEP**	**0.60**^**b**^	**0.40**	**0.88**	0.78	0.56	1.10	**0.64**^**a**^	**0.39**	**1.04**
**MMP**	0.79	0.59	1.05	0.93	0.74	1.17	0.86	0.66	1.13
**MBzP**	**0.76**^**a**^	**0.55**	**1.04**	1.11	0.84	1.45	0.77	0.52	1.15
**MiBP**	0.78	0.50	1.20	0.95	0.65	1.39	1.05	0.59	1.88
**OH-MiBP**	0.97	0.73	1.30	1.08	0.85	1.38	0.98	0.60	1.62
**MnBP**	0.71	0.46	1.10	0.92	0.62	1.37	0.71	0.43	1.17
**OH-MnBP**	0.91	0.68	1.22	1.11	0.84	1.48	**0.72**^**a**^	**0.49**	**1.04**
**MEHP**	0.81	0.61	1.08	0.86	0.64	1.15	0.76	0.52	1.10
**5OH-MEHP**	0.70	0.41	1.21	0.86	0.53	1.39	0.82	0.52	1.30
**5oxo-MEHP**	0.69	0.40	1.20	0.83	0.49	1.40	0.78	0.47	1.28
**5cx-MEPP**	0.74	0.41	1.34	0.72	0.41	1.28	0.70	0.40	1.24
**OH-MiNP**	1.15	0.78	1.71	1.07	0.76	1.50	1.14	0.69	1.88
**oxo-MiNP**	1.15	0.78	1.69	1.04	0.74	1.47	1.08	0.67	1.74
**cx-MiNP**	1.31	0.88	1.97	0.98	0.68	1.42	1.10	0.63	1.94
**OH-MiDP**	1.13	0.73	1.73	1.41	0.91	2.19	0.76	0.45	1.28
**oxo-MiDP**	1.36	0.93	1.98	1.40	0.92	2.14	0.82	0.54	1.25
**cx-MiDP**	**1.81**^**b**^	**1.19**	**2.74**	**1.65**^**b**^	**1.05**	**2.61**	0.74	0.44	1.22
**BPA**	0.79	0.57	1.10	1.18	0.87	1.60	1.25	0.84	1.88

Adjusted for age (years), BMI (kg/m^2^), creatinine (mg/dl); OR: Odds Ratio.

95% CI: 95% confidence interval of the OR

a: p ≤ 0.1

b: p ≤ 0.05

c: PD scale ≥ 3 = definitely started and higher = yes

d: onset of menarche = yes

For boys, the single PD scales for facial hair growth and voice change showed only in the third follow-up study a small number of positive answers for “definitely started” or higher (Table 2). Therefore, we calculated logistic regression models for these two items from the third follow-up study (Table 6). For pubic hair growth a gradual increase in the number of positive answers for “definitely started” and higher was detected, so we conducted a GEE analyses. Compared with the overall PD scores, we detected similar results when using the single PD scales for pubic hair growth, facial hair growth and voice change in the binomial analyses. The LMW metabolite concentrations and the DEHP and DiNP metabolite levels were positively associated with facial hair growth (e.g. OR’s_DEHP_ 2.1–3.1; OR’s_DiNP_ 1.03–1.4; OR_MEP_ 1.63). Voice change and pubic hair growth were almost negatively associated with the metabolite concentrations with significant associations between the DEHP metabolites and pubic hair growth (OR’s 0.47–0.71). Voice change and pubic hair growth appeared to be more affected by the metabolite concentrations than facial hair growth. The BPA concentrations showed no clear relationship with the single PD scales.

**Table 6:** Logistic regression and binomial GEE analyses of single PD scales with phthalate metabolites and BPA concentrations (ln- μg/l) in boys (N = 210).

Metabolite	Facial hair growth^c^ (yes vs. no)^e^			Voice change^c^ (yes vs. no)^e^				Pubic hair growth^d^ (yes vs. no)^e^		
	OR	95% CI			OR	95% CI		OR	95% CI	
**MEP**	**1.63**^**a**^	**0.95**	**2.79**		0.26	0.05	1.35	0.83	0.55	1.25
**MMP**	0.98	0.67	1.43		0.82	0.39	1.72	0.83	0.63	1.08
**MBzP**	1.04	0.61	1.80		0.67	0.21	2.13	0.92	0.59	1.43
**MiBP**	1.12	0.55	2.26		0.32	0.07	1.41	1.17	0.69	1.99
**OH-MiBP**	1.18	0.64	2.15		0.64	0.32	1.29	0.97	0.67	1.40
**MnBP**	1.10	0.51	2.36		0.57	0.13	2.56	0.80	0.43	1.48
**OH-MnBP**	1.04	0.57	1.90		0.68	0.25	1.83	0.74	0.46	1.20
**MEHP**	**2.10**^**b**^	**1.21**	**3.64**		1.48	0.60	3.62	**0.71**^**b**^	**0.51**	**0.99**
**5OH-MEHP**	**2.45**^**b**^	**1.18**	**5.07**		0.89	0.23	3.47	**0.58**^**b**^	**0.34**	**0.98**
**5oxo-MEHP**	**3.11**^**b**^	**1.42**	**6.82**		0.78	0.19	3.23	**0.58**^**a**^	**0.32**	**1.04**
**5cx-MEPP**	**2.87**^**b**^	**1.30**	**6.33**		0.97	0.20	4.63	**0.47**^**b**^	**0.26**	**0.83**
**OH-MiNP**	1.40	0.73	2.69		1.00	0.43	2.34	0.78	0.49	1.26
**oxo-MiNP**	1.16	0.63	2.14		0.74	0.25	2.23	0.76	0.49	1.19
**cx-MiNP**	1.03	0.50	2.11		0.32	0.06	1.67	0.76	0.48	1.19
**OH-MiDP**	1.11	0.61	2.02		1.35	0.48	3.80	0.76	0.47	1.20
**oxo-MiDP**	0.86	0.46	1.63		1.72	0.53	5.55	0.86	0.51	1.45
**cx-MiDP**	0.94	0.46	1.92		0.39	0.09	1.63	0.83	0.48	1.43
**BPA**	1.08	0.59	2.00		1.07	0.36	3.16	0.73	0.47	1.14

Adjusted for age (years), creatinine (mg/dl), BMI (kg/m^2^); OR = Odds Ratio; 95% CI = 95% confidence interval of the OR.

a: p ≤ 0.1

b: p ≤ 0.05

c: calculated by multiple logistic regression for the third follow-up study

d: calculated by binomial GEE for 3 follow-up studies

e: PD scale ≥ 3 = definitely started and higher = yes

## Discussion

Although the epidemiological literature is expanding, the current evidence does not show a definite link between phthalate exposure and reproductive outcomes in humans. This is possibly due to small sample sizes, variability of concurrent and prenatal exposures, and only few measures in relevant exposure windows [26–28]. Several more recent epidemiological studies report on relationships of current phthalate metabolite levels with the pubertal development of adolescents, although the results are still inconsistent.

Frederiksen et al. [9] observed delayed pubarche but not delayed thelarche in Danish girls with increasing current phthalate metabolite concentrations. Similarly, Wolff and co-workers [11] demonstrated delayed pubic hair development with HMW phthalates and delayed breast development with MBzP in American girls. Su et al. [29] detected reduced uterus size with higher MEHP and ∑DEHP concentrations and a negative association between bone age/chronological age ratio and MBzP exposure. The studies from Tsai et al. and Xie et al. [30, 14] showed delayed growth characteristics in youngsters with increasing pre-pubertal phthalate exposures in children. Results from Watkins et al. [31] point in the same direction: Phthalate metabolites and BPA were associated with metabolism biomarkers (e.g. insulin or leptin excretion) at age 8–14 years in patterns that varied by sex, pubertal status, and exposure timing. We detected a delay of the pubertal development in girls, mainly for the pre-pubertal exposure with DEHP, DEP, DnBP, and BBzP metabolites. Furthermore, we detected non-linear effects of the DEHP metabolites and BPA on the PD scores in girls. These observations support the idea that non-monotonic dose-response curves are a fundamental feature of hormones and therefore also of endocrine disrupting agents (EDC’s) [32].

Following publications show different results: Zhang et al. [33] reported that MnBP exposure is associated with delayed pubic hair development in boys, and the DEHP metabolites are associated with earlier menarche onset in girls. Data from Mouritsen et al. [13] indicate that exposure to DBP isomers (in girls) and MBzP (in boys) are negatively associated with adrenal androgen levels, and MBzP is associated with earlier age at pubarche in boys. Watkins and colleagues reported accelerated pubertal development [10]: MEP and increased odds of reaching menarche and MnBP, and increased odds of having a Tanner stage >1 for both breast and pubic hair development. Mieritz et al. [34] did not observe associations of current phthalate exposures with age at pubertal onset, serum testosterone levels and the presence of gynaecomastia in Danish boys. However, we found positive associations of the pubertal development with the DEHP and DEP metabolites in boys, specifically for facial hair growth, and only small relationships with the other phthalates.

An involvement of the current BPA exposure on the pubertal development in girls was shown by Durmaz et al. [35] (precocious puberty) and McGuinn et al. [12] (delayed menarche). Ferguson and co-workers [36] observed no association with puberty and childhood phthalate exposure, but some phthalates and also BPA were associated with increased sex hormone binding globulin and decreased testosterone levels. In our study we observed relationships between the BPA exposure and lower PD scores in girls but not in boys.

EDC’s exert their effects by mimicking, antagonizing or altering steroidal actions. Decreased steroidogenesis and circulating testosterone concentrations are thought to be central to phthalate-induced reproductive toxicity [37, 26]. As phthalates are considered to be anti-androgenic compounds, delayed appearance of pubic hair is not unexpected. Similarly, while phthalates are weakly estrogenic, they may also be able to affect breast development [27]. Martinez-Arguelles et al. [38] reviewed rodent and *in vitro* studies for developmental impacts due to prenatal and current DEHP exposures. The authors found that in comparison to males, females are more resistant to the endocrine disrupting effects of prenatal DEHP. However, decreased levels of estradiol are a constant finding immediately after exposure in females, as is the decreased expression of aromatase.

Pubertal timing is dependent on many factors such as genetic effects [39], physical activity, fat mass, height, insulin resistance, psychological factors, nutrition [40], prenatal environmental exposures and/or exposures earlier in life that may explain the differences in the reported studies. Additionally, inconsistent results among the epidemiological studies may also be due to the broad variety of outcome measures.

The German Human Biomonitoring (HBM) commission provides toxicologically derived threshold values for the DEHP metabolites in humans [41–42]. In our study population the DEHP metabolite concentrations were almost all below the HBM-I values (indicating no relevant risk). However, even at low levels, exposure to environmental EDC’s may interfere with normal endocrine processes. This is particularly the case during critical periods of the development, such as intrauterine, perinatal, and juvenile or puberty periods, when organisms are more sensitive to hormonal disruption compared to other periods [43]. Furthermore, up to now it is unknown whether different phthalates act together, maybe combined with other environmental EDC’s (e.g. PCB or paracetamol), or whether a single compound may influence the pubertal timing.

Taken together, several studies point in the direction of affecting the pubertal development in girls by current or pre-pubertal phthalate exposure. However, the known anti-androgenic effects of phthalates are more likely linked to prenatal exposures during pregnancy and a delay of the pubertal development in boys. In our study, the pre-pubertal exposures to phthalates and BPA in boys were not clearly associated with the summary PD scores. Furthermore, the current literature does not provide consistent data for favoring the hypothesis that the phthalate exposure during childhood affects the puberty in boys [26].

### Limitations

Excretion half-lives of phthalates and BPA are below 24 hours and long-term exposure estimations are limited. Personal variations and the day-to-day variability of phthalate/BPA excretion complicate the estimation of long-term exposures, or exposures in time windows deemed relevant [13, 44–47]. Therefore, careful consideration is required when interpreting epidemiological study results [48]. We collected first morning urine samples and the time of sampling was similar across our samples. Moreover, we showed significant correlations of phthalate metabolites measured twice in a two-year sampling period in a subsample of children from the Duisburg cohort [16] (Fig 1), implicating similar exposure characteristics in the six- to seven- and eight- to nine-year-old children. Nevertheless, the single exposure measures in our baseline study cannot account for the within-individual variation and the day-to-day-variability of phthalate and BPA exposures and are a limitation of our study.

Exposure sources of certain phthalates and BPA might be identical or related to exposure sources of other chemicals (such as parabens, environmental phenols, sunscreens etc.) [49, 50] which were not determined in this study. Co-exposures to other known or unknown chemicals and combined exposures have not been accounted for in this study but are major challenges of both future toxicological and epidemiological studies [51–52].

Watzlawick [20] and Bond et al. [53] validated the PD scale questionnaire and their results were in moderate to good agreement with the physical examinations of the pubertal stages according to Marshall and Tanner [54–55]. Unfortunately, we were not able to validate the quality of the PD scale questionnaire by examinations of the pubertal development according to Marshall and Tanner [54–55] who developed the so-called “gold standard” by physical examinations, and this is a limitation of the study.

Rasmussen and colleagues [56] examined the quality of the self-assessment of pubertal stages (illustrations of pubic hair, genital and girl’s breast development). They observed that girls tended to underestimate and boys to overestimate their pubertal stage compared with physical examinations but this differs across populations and studies [56–57]. We also detected overestimation of the pubertal development, particularly in boys, because more boys than girls answered on a higher scale one or two years earlier. For the boys, this fact mainly influenced the poorer reliability of the PD scales in the baseline and first follow-up studies compared to the girls (S3 Table). Rasmussen et al. [56] concluded that a self-report of puberty outcomes such as onset of pubarche or thelarche is sufficient to distinguish between pre-puberty and puberty when drawings of the pubertal stages were used. Compared with the PD scales this is a quite different method and the PD scales cannot easily be compared with drawings of the pubertal stages. Therefore, there was no direct relation to Rasmussen’s assessment of the pubertal process.

The validity and reliability of the imputed PD scale data, tested by Cronbach’s alpha test, was moderate with alpha coefficients of approximately 0.4 to 0.6 for girls (S3 Table). Alpha values > 0.7 are generally acceptable. However, lower values should be sufficient for the low number of items used because the alpha value correlates with the number of items. When the number of items is < 4, then a lower alpha-value would be acceptable [23, 58]. For boys, the values were between 0.25 and 0.56, reflecting poorer reliability. However, our longitudinal study design allowed for detecting overestimations for each child, whereas in most studies the pubertal development was recorded only once. This allowed us to account for the overestimations prior to the imputation process.

Nevertheless, our longitudinal recording with the PD scale questionnaire was in good agreement with the expected development in boys and girls, and was satisfactory to determine the rising process of the puberty. The PD scale questionnaire may be a good alternative to physical examinations in epidemiological studies due to higher participation rates and lower costs but this instrument may not overcome the advantage of physical examinations.

## Conclusion

This is the first environmental epidemiological study that longitudinally assessed the pubertal development with the PD scale questionnaire during a four-year study period. The PD questionnaire will be a good tool in epidemiological studies compared to time and cost consuming physical examinations. Our data indicate that the pre-pubertal exposures to some phthalates and BPA at low background levels might be associated with the pubertal timing, particularly in girls. Associations in boys were inconsistent and not necessarily in line with the known anti-androgenicity of some phthalates during prenatal exposure. Assessing suitable surrogates for long-term and cumulative exposure scenarios and determining the critical time windows of exposures during childhood should be addressed to future studies.

## Supporting information

S1 Table Full names of the phthalates and their metabolites. (DOCX)

S2 Table Key questions of the PD scale questionnaire (children’s version). (DOCX)

S3 Table Correlation coefficients (Cronbach’s alpha) of the PD scale data before and after imputation of missing values. (DOCX)

S4 Table Multinomial GEE regression models of original PD scores (from first, second, and third follow-up) and phthalate/BPA concentrations (ln-μg/l). (DOCX)

S5 Table Correlation coefficients (Spearman’s r) between phthalate metabolites and BPA (μg/l). (DOCX)
