# Supplementary material for: Pre-pubertal exposure with phthalates and bisphenol A and pubertal development
Source: PLoS One. 2017 Nov 20;12(11):e0187922. doi: 10.1371/journal.pone.0187922 (PMC5695814; doi:10.1371/journal.pone.0187922)
Supplement: S1 Table — (DOCX) [file pone.0187922.s001.docx]

**S1 Table. Full names of the phthalates and their metabolites.**

| **Parent phthalate** | **Primary metabolite**  **(monoester of the parent compound)** | **Secondary metabolite**  **(oxidized metabolites of the monoester)** |
| --- | --- | --- |
| **HMW phthalates** | | |
| **Di-(2-ethylhexyl) phthalate (DEHP)** | Mono-2-ethylhexyl) phthalate (MEHP) | Mono-(2-ethylhexyl-5-hydroxyhexyl) phthalate (5OH-MEHP) |
|  |  | Mono-(2-ethylhexyl-5-oxohexyl) phthalate (5oxo-MEHP) |
|  |  | Mono-(5-carboxy-2-ethylpentyl) phthalate (5cx-MEPP) |
| **Di-isononyl phthalate (DiNP)** | - | 7OH-Mono-methyloctyl phthalate (OH-MiNP) |
|  |  | 7oxo-Mono-methyloctyl phthalate (oxo- MiNP) |
|  |  | 7carboxy-Mono-methylheptyl phthalate (cx-MiNP) |
| **Di-isodecyl phthalate (DiDP) and Di-propyl-heptyl phthalate (DPHP)^a^** | - | 6OH-Mono-propylheptyl phthalate (OH-MiDP) |
|  |  | 6oxo-Mono-propylheptylphthalate (oxo-MiDP) |
|  |  | Mono(2,7-methyl-7carboxy-heptyl) phthalate (cx-MiDP) |
| **Di-*n*-ocytl phthalate (DnOP)** | Mono-n-ocytl phthalate (MnOP) | - |
| **LMW phthalates** | | |
| **Di-methyl phthalate (DMP)** | Mono-methyl phthalate MMP | - |
| **Diethyl phthalate (DEP)** | Monoethyl phthalate (MEP) | - |
| **Di-cyclohexyl phthalate (DCHP)** | Mono-cyclohexyl phthalate (MCHP) | - |
| **Di-*n*-pentyl phthalate (DnPeP)** | Mono-n-pentyl phthalate (MnPeP) | - |
| **Butyl benzyl phthalate (BBzP)** | Mono-benzyl phthalate (MBzP) | - |
| **Di-isobutyl phthalate (DiBP)** | Mono-isobutyl phthalate (MiBP) | 2OH-Mono-isobutyl phthalate (2OH-MiBP) |
| **Di-*n*-butyl phthalate (DnBP)** | Mono-*n*-butyl phthalate (MnBP) | 3OH-Mono-n-butyl phthalate (3OH-MnBP) |
| **Metabolites common to several parent compounds** | | |
| **DnBP, DnOP, DnPeP, DiDP, DiNP** | - | 3-carboxyl-mono-propyl phthalate (MCPP)^b^ |

Differing metabolite abbreviations used in the some other studies are as follows: MCPP: 3cx-MPP; 5OH-MEHP: MEHHP; 5oxo-MEHP: MEOHP; 5cx-MEPP: MECPP; cx-MiNP: MCOP; cx-MiDP: MCNP.

a: HPLC-MS/MS methods cannot distinguish between DiDP and DPHP metabolites.

b: MCPP is a metabolite of several HMW and LMW phthalates (currently known: DnBP, DnPeP, DnOP, DiNP, DiDP).
