# Supplementary material for: Pre-pubertal exposure with phthalates and bisphenol A and pubertal development
Source: PLoS One. 2017 Nov 20;12(11):e0187922. doi: 10.1371/journal.pone.0187922 (PMC5695814; doi:10.1371/journal.pone.0187922)
Supplement: S2 Table — (DOCX) [file pone.0187922.s002.docx]

**S2 Table. Key questions of the PD scale questionnaire (children’s version).**

|  | **Question** | **Answer** |
| --- | --- | --- |
| **Form for girls and boys:** | Would you say that your growth in height has accelerated: | - not yet happened (1 point) - barely started (2 points) - definitely started (3 points) - seems complete (4 points) - I don’t know |
|  | Would you say that your body hair has started to grow: |  |
|  | Have you noticed any skin changes, especially pimples? |  |
| **Form for boys:** | Have you noticed a deepening of your voice? |  |
|  | Have you begun to grow hair on your face? |  |
| **Form for girls:** | Have you noticed that your breasts have begun to bud? |  |
|  | Have you begun to menstruate (started to have your period)? | No/yes |
|  | If yes, please give the date when you started to menstruate: | Date/year |
