# Supplementary material for: Pre-pubertal exposure with phthalates and bisphenol A and pubertal development
Source: PLoS One. 2017 Nov 20;12(11):e0187922. doi: 10.1371/journal.pone.0187922 (PMC5695814; doi:10.1371/journal.pone.0187922)
Supplement: S3 Table — (DOCX) [file pone.0187922.s003.docx]

**S3 Table. Correlation coefficients (Cronbach’s alpha) of the PD scale data before and after imputation of missing values.**

|  | **Girls** | | **Boys** | | **Both** | |
| --- | --- | --- | --- | --- | --- | --- |
| **Examination period** | **Before (N=96)** | **After (N=198)** | **Before (N=80)** | **After (N=210)** | **Before (N=176)** | **After (N=408)** |
| **Baseline** | 0.44 | 0.43 | 0.46 | 0.56 | 0.41 | 0.46 |
| **First follow-up** | 0.48 | 0.49 | 0.72 | 0.25 | 0.53 | 0.42 |
| **Second follow-up** | 0.58 | 0.55 | 0.48 | 0.34 | 0.52 | 0.48 |
| **Third follow-up** | 0.70 | 0.63 | 0.51 | 0.45 | 0.63 | 0.53 |
