# Supplementary material for: Pre-pubertal exposure with phthalates and bisphenol A and pubertal development
Source: PLoS One. 2017 Nov 20;12(11):e0187922. doi: 10.1371/journal.pone.0187922 (PMC5695814; doi:10.1371/journal.pone.0187922)
Supplement: S4 Table — (DOCX) [file pone.0187922.s004.docx]

**S4 Table. Multinomial GEE regression models of original PD scores (from first, second, and third follow-up) and phthalate/BPA concentrations (ln-µg/l).**

|  | **Boys original data (N=79)** | | | **Girls original data (N=96)** | | |
| --- | --- | --- | --- | --- | --- | --- |
| **Substance (µg/l –ln-transformed)** | **β** | **95% CI** | | **β** | **95% CI** | |
| **MEP** | 0.008 | -0.224; | 0.241 | -0.130 | -0.310; | 0.050 |
| **MMP** | -0.095 | -0.213; | 0.024 | 0.001 | -0.115; | 0.117 |
| **MBzP** | -0.135 | -0.333; | 0.063 | -0.044 | -0.231; | 0.145 |
| **MiBP** | -0.055 | -0.225; | 0.115 | -0.124 | -0.358; | 0.110 |
| **OH-MiBP** | -0.033 | -0.119; | 0.054 | -0.019 | -0.227; | 0.188 |
| **MnBP** | -0.113 | -0.349; | 0.123 | -0.092 | -0.318; | 0.133 |
| **OH-MnBP** | -0.085 | -0.218; | 0.049 | **-0.182^b^** | **-0.342;** | **-0.022** |
| **MEHP** | **0.116^a^** | **-0.005;** | **0.236** | -0.169 | -0.375**;** | 0.038 |
| **OH-MEHP** | 0.024 | -0.209; | 0.256 | -0.125 | -0.373**;** | 0.123 |
| **oxo-MEHP** | 0.034 | -0.234; | 0.301 | -0.156 | -0.421**;** | 0.109 |
| **cx-MEHP** | 0.044 | -0.248; | 0.336 | -0.185 | -0.467**;** | 0.096 |
| **OH-MiNP** | 0.129 | -0.071; | 0.328 | 0.122 | -0.030; | 0.273 |
| **oxo-MiNP** | 0.058 | -0.082; | 0.198 | 0.047 | -0.169**;** | 0.264 |
| **cx-MiNP** | 0.005 | -0.179; | 0.189 | 0.118 | -0.130; | 0.365 |
| **OH-MiDP** | 0.204 | -0.043; | 0.451 | 0.003 | -0.216; | 0.221 |
| **oxo-MiDP** | 0.185 | -0.077; | 0.448 | 0.017 | -0.157; | 0.191 |
| **cx-MiDP** | 0.187 | -0.164; | 0.538 | 0.128 | -0.058; | 0.313 |
| **BPA** | -0.008 | -0.213; | 0.197 | 0.012 | -0.167; | 0.192 |

95% CI: 95% confidence interval; β: regression coefficient;

a: p ≤ 0.1; b: p ≤ 0.05; adjusted for: BMI, age, urinary creatinine (mg/dl)
