# Supplementary material for: Pre-pubertal exposure with phthalates and bisphenol A and pubertal development
Source: PLoS One. 2017 Nov 20;12(11):e0187922. doi: 10.1371/journal.pone.0187922 (PMC5695814; doi:10.1371/journal.pone.0187922)
Supplement: S5 Table — (DOCX) [file pone.0187922.s005.docx]

**S5 Table. Correlation coefficients (Spearman’s r) between phthalate metabolites and BPA (µg/l).**

| **Spear-man's r** |  | | | | | | | | |
| --- | --- | --- | --- | --- | --- | --- | --- | --- | --- |
| **p-value** |  | | | | | | | | |
|  | **Sum DEHP** | **MEP** | **MMP** | **MBzP** | **Sum DiNP** | **Sum DiDP** | **Sum DiBP** | **Sum DnBP** | **BPA** |
| **Sum DEHP** |  | 0.442 | 0.186 | 0.585 | 0.641 | 0.570 | 0.592 | 0.608 | 0.457 |
|  |  | <.0001 | <.0001 | <.0001 | <.0001 | <.0001 | <.0001 | <.0001 | <.0001 |
| **MEP** | 0.442 |  | 0.334 | 0.442 | 0.338 | 0.297 | 0.563 | 0.545 | 0.274 |
|  | <.0001 |  | <.0001 | <.0001 | <.0001 | <.0001 | <.0001 | <.0001 | <.0001 |
| **MMP** | 0.186 | 0.334 |  | 0.222 | 0.221 | 0.205 | 0.319 | 0.282 | 0.155 |
|  | <.0001 | <.0001 |  | <.0001 | <.0001 | <.0001 | <.0001 | <.0001 | 7E-04 |
| **MBzP** | 0.585 | 0.442 | 0.222 |  | 0.481 | 0.463 | 0.545 | 0.617 | 0.309 |
|  | <.0001 | <.0001 | <.0001 |  | <.0001 | <.0001 | <.0001 | <.0001 | <.0001 |
| **Sum DiNP** | 0.641 | 0.338 | 0.221 | 0.481 |  | 0.730 | 0.452 | 0.433 | 0.354 |
|  | <.0001 | <.0001 | <.0001 | <.0001 |  | <.0001 | <.0001 | <.0001 | <.0001 |
| **Sum DiDP** | 0.570 | 0.297 | 0.205 | 0.463 | 0.730 |  | 0.446 | 0.447 | 0.370 |
|  | <.0001 | <.0001 | <.0001 | <.0001 | <.0001 |  | <.0001 | <.0001 | <.0001 |
| **Sum DiBP** | 0.592 | 0.563 | 0.319 | 0.545 | 0.452 | 0.446 |  | 0.719 | 0.365 |
|  | <.0001 | <.0001 | <.0001 | <.0001 | <.0001 | <.0001 |  | <.0001 | <.0001 |
| **Sum DnBP** | 0.608 | 0.545 | 0.282 | 0.617 | 0.433 | 0.447 | 0.719 |  | 0.384 |
|  | <.0001 | <.0001 | <.0001 | <.0001 | <.0001 | <.0001 | <.0001 |  | <.0001 |
| **BPA** | 0.46 | 0.27 | 0.15 | 0.31 | 0.35 | 0.37 | 0.36 | 0.38 |  |
|  | <.0001 | <.0001 | 7E-04 | <.0001 | <.0001 | <.0001 | <.0001 | <.0001 |  |
